# Reliability and validity of the Nepean Beliefs Scale for delusions and overvalued ideas in chronic schizophrenia: analysis of a preliminary pilot study

**DOI:** 10.3389/fpsyt.2023.1298429

**Published:** 2023-12-07

**Authors:** Takuma Ishigaki, Takeshi Shimada, Hiroki Tanoue, Naoki Yoshinaga, Yuki Nishiguchi, Ryotaro Ishikawa, Masahito Hosono

**Affiliations:** ^1^Graduate School of Arts and Sciences, The University of Tokyo, Meguro, Japan; ^2^Department of Rehabilitation, Mental Support Soyokaze Hospital, Ueda, Japan; ^3^Faculty of Medicine, University of Miyazaki, Miyazaki, Japan; ^4^Faculty of Education, Chiba University, Chiba, Japan; ^5^Faculty of Psychology and Sociology, Taisho University, Tokyo, Japan

**Keywords:** Nepean Beliefs Scale (NBS), schizophrenia, delusions, overvalued ideas, validity, reliability

## Abstract

**Introduction:**

The Nepean Beliefs Scale by Brakoulias et al. is an interview-based multidimensional instrument that measures pathological beliefs in various psychiatric disorders. This study examined the reliability and validity of Nepean Beliefs Scale (NBS) for delusions and overvalued ideas in patients with chronic-phase schizophrenia. Methods: Multiple raters at two healthcare settings examined the beliefs of 28 individuals with schizophrenia using the NBS. Concurrently, PANSS, PDI-21, BCIS, PHQ-9 and GAD-7 were administered.

**Results:**

The NBS had high reliability and correlation with relevant scales.

**Discussion:**

The NBS was found to have sufficient reliability and validity for assessing the pathological beliefs of patients with chronic schizophrenia. Although NBS is an easy-to-instruct instrument, it should be noted that appropriate explanations and examples should be added to instructions to obtain reliable responses from patients with chronic schizophrenia.

## 1 Introduction

The Nepean Beliefs Scale (NBS) is an objective rating scale developed by Brakoulias et al. (1) at the University of Sydney, that examines obsessive thoughts and overvalued ideas. An overvalued idea is cross-diagnostic symptom that can occur in many psychiatric disorders. However few scales exist to quantitatively measure it. The Brown Assessment of Beliefs Scale Adult Version (2) and the Overvalued Ideas Scale (3) are pioneering scales; the NBS was developed as a simplified version of these scales. The NBS uses a 0–4 five-point scale to assess five items of overvalued ideas: conviction, fixity, fluctuation, resistance, and reasonableness of belief through an interview.

Although initially developed to examine obsessions, NBS has been suggested to be reliable and valid when used to assess overvalued ideas in anorexia nervosa (4) and delusions and pathological beliefs in acute psychosis (5). In particular, the subjects in Rajendran et al.’s study were diverse, including those with schizophrenia, schizoaffective disorder, bipolar affective disorder, delusional disorder, substance/drug-induced psychotic disorder, and short-term psychotic disorder. High reliability and validity of NBS for delusions and pathological beliefs provide evidence of the cross-diagnostic validity of this scale.

However, delusions in chronic-phase schizophrenia are presumed to be psychosocially modified because of the long course of the illness, and it is unclear whether the results would be the same as those obtained in acute-phase psychosis. If the NBS is reliable and valid when used with chronic schizophrenia, it will not only broaden the cross-diagnostic use of the NBS but may also be validated in longitudinal studies that assess long-term treatment effects. Furthermore, it may be helpful to examine the effects of cognitive behavioral therapy, which can be used to modify pathological beliefs regarding chronic conditions.

This multicenter study was a preliminary investigation of the reliability and validity of NBS for delusions in chronic schizophrenia. Clinical studies of the NBS have examined its validity by correlating it with existing overvalued idea scales such as the Brown Assessment of Beliefs Scale and the Overvalued Ideas Scale, but unfortunately Japanese versions do not exist. Therefore, we examined the validity of the Japanese version of the NBS by correlating it with self-administered scales that examine delusions and delusional ideation. In addition, the characteristics of delusions in chronic schizophrenia, as reflected in the NBS, are discussed based on a comparison with previous studies.

## 2 Methods

### 2.1 Preparation of the Japanese version of the NBS

After obtaining permission from the original author (Prof. Vlasios Brakoulias), the Japanese version of the NBS was prepared through Japanese translation, back-translation, and confirmation by the original author. Similar to the original version, the NBS consists of five items evaluated a five-point scale.

### 2.2 Participant selections

Outpatients aged between 20 and 65 years who had been diagnosed with schizophrenia and who regularly attended two hospitals, the Mental Support Soyokaze Hospital and Wakakusa Hospital, were included in the recruitment process. Medical history, sex, educational background, employment status, residential status, and ethnicity/race were excluded. These participants were recruited for a larger research project includes this study; in the recruited outpatients, people with a disease duration of more than 10 years were included in this study. Patients were recruited with the permission of their attending physicians after an ethical review at two hospitals affiliated with the research collaborators. A brief explanatory document was presented, and if the participants expressed interest, they were given a more detailed explanatory document and a consent form. Applicants were again evaluated using the Structured Clinical Interview for DSM-5 Disorders Research Version (SCID-5-RV) (6) and were considered study participants if they met the diagnostic criteria for schizophrenia. The exclusion criteria were coexisting obsessive-compulsive disorder, bipolar disorder, depressive disorder, neurodevelopmental disorders, and substance-related and addictive disorders. These disorders were used as exclusion criteria in the present study for comparison with data on schizophrenia in a near future study.

### 2.3 Study participants

Thirty-three individuals were recruited and 29 who met the requirements became study participants, one of whom refused to be retested; all analyses were performed on data from the remaining 28 participants.

### 2.4 Ethical review and costs

This study was approved by the Ethical Review Committee for Experimental Research on Human Subjects of Graduate School of Arts and Sciences, the University of Tokyo (Project No. 791–5). The costs of this research were covered by a JSPS Grant-in-Aid for Scientific Research (Project No. 22 K03096, PI: Takuma Ishigaki).

### 2.5 Scales used in the survey

The scales used, except the NBS, include The Positive and Negative Syndrome Scale (PANSS) (7), which is based on interviews with patients by trained assessors using a seven-point scale, with a score of 7 indicating the most severe illness. Only the delusion item was used in the present study. The 21-item version developed by Peters et al. The Delusions Inventory [PDI-21; (8, 9)], which lists as questions is a self-administered scale that assesses the number of delusional thoughts and the degree of preoccupation, conviction, and distress of such thoughts on a five-point scale: The Beck Cognitive Insight Scale (BCIS) (10, 11) is a self-administered, four-point scale (“not agree at all = 0 point” to “agree completely = 3 point”) with 15 items of insight into disease. The Patient Health Questionnaire-9 [PHQ-9; (12)] is a self-administered measure of the degree of depression, with each of nine items rated on a scale from “not at all = 1 point” to “every day = 4 point” for how often they are bothered. The Generalized Anxiety Disorder-7 [GAD-7; (13)] is a self-administered measure of anxiety, with each of the seven items rated on a scale from “not at all = 0” to “almost every day = 3.” The Japanese versions of the PHQ-9 and the GAD-7 are provided free of charge to members of the Japanese Society of Anxiety and Related Disorders.

### 2.6 Survey method

A 10-min practice video was created to ensure that all research collaborators watched and practiced with the NBS before beginning the survey. Because the participants were relatively old and chronically ill, it was expected that they would have varying degrees of cognitive dysfunction; although the NBS is inherently simple and easy to answer, it was possible that they would have difficulty understanding the questions and selecting answers. Therefore, we added a rating for ease of response, giving two points to those who were able to answer the questions using only the questions and options provided in the NBS, and one point to those who needed to provide examples or explanations for all items in the NBS. A score of 0.2 was subtracted for each item for which an example or explanation was provided. In other words, there were 2.0, 1.8, 1.6, 1.4, 1.2, and 1.0 points.

The NBS was rated by raters with at least 5 years of psychiatric practice. To examine the inter-rater reliability, the first survey was conducted simultaneously by two raters at each hospital (Raters 1 and 2). The second survey was conducted 2–4 weeks after the first evaluation by Rater 1. Raters 1 and 2 were psychiatric nurses or occupational therapists. The evaluation using the delusion item of the PANSS was also conducted by Rater 1, who received training.

If a participant had multiple delusions, they were asked to select the one that interfered the most with their life or caused the most distress, which was the subject of the evaluation.

### 2.7 Data analysis

SPSS® ver. 28 was used for all analyses.

## 3 Results

### 3.1 Attributes of participants

All participants were Japanese nationals who had grown up in Japan. The age, sex, duration of illness, and daily dose of medication of the participants were shown in Table 1.

**Table 1:** Descriptive statistics of all scales, socio-geographic and clinical data.

	Mean	SD	Minimum	Maximum
NBS: total score	14.93	4.66	3	20
NBS: item 1	3.00	1.12	1	4
NBS: item 2	2.86	1.30	0	4
NBS: item 3	2.96	1.07	0	4
NBS: item 4	3.00	1.12	0	4
NBS: item 5	3.11	0.83	1	4
rating for ease of response	1.21	0.30	1	2
PANSS: delusion item	4.68	1.19	3	7
PDI: number of “YES” items	8.00	5.18	0	18
PDI: preoccupation	32.29	21.48	0	70
PDI: conviction	33.79	22.96	0	82
PDI: distress	30.64	20.24	0	32
BCIS: total score	20.21	5.56	0	32
BCIS: self-reflectiveness	9.32	4.46	0	20
BCIS: self-certainty	10.89	4.04	0	16
PHQ-9	8.54	4.53	2	17
GAD-7	9.00	5.77	0	19
Age: all patients	48.79	8.78	30	62
Age: Men (*N* = 12)	45.85	9.86	30	62
Age: Women (*N* = 16)	51.75	7.06	38	61
Duration of illness	27.71	7.16	14	41
Medication (chlorpromazine equivalent per day)	855.11	636.40	100	1,000

### 3.2 Examples of beliefs that were evaluated

Examples of participants’ beliefs are given below: “Poison has been put in my medicine without my knowledge, and it makes me sick,” “The neighbor is breaking into my room and spreading powder poison, and even though I lock the door and blind the window, he still gets in, and it keeps me up at night and makes me feel sick,” “Someone has been cutting my hair or burning it when I sleep at night, and when I ask them about it, they say they do not know and are lying,” “I am being told bad things about myself on TV,” “I am the president of a company, but salary has not been paid for a long time, and in time I will receive a large sum of money,” “I am being watched by others, so I must not move,” and “My grandmother is manipulating me and trying to make me look bad.”

### 3.3 Descriptive statistics of scales and correlation analysis

Table 1 presented the descriptive statistics for all scales.

**Figure 1:**
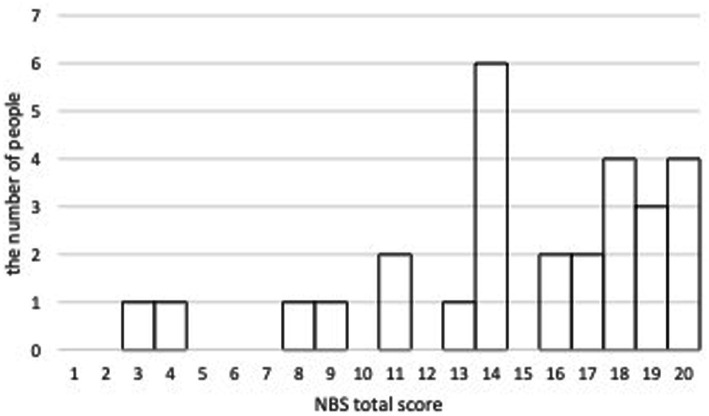
The histogram of the total score of NBS.

The mean NBS total score from the first evaluation by Rater 1 was 14.93 (SD = 4.66). The NBS total score averaged 15.08 (SD = 4.83) for men and 14.81 (SD = 4.68) for women, with no significant differences between the sexes. The histogram of the total scores of NBS from the first evaluation by Rater 1 was shown in Figure 1.

There were no significant correlations between the total score of NBS and age, duration of illness, or the amount of medication administrated.

The correlations between each scales showed Table 2.

**Table 2:** Correlations between NBS score and other scales.

	NBS total	NBS item 1	NBS item 2	NBS item 3	NBS item4	NBS item5
NBS: item 1	0.900^**^					
NBS: item 2	0.936^**^	0.865^**^				
NBS: item 3	0.920^**^	0.832^**^	0.849^**^			
NBS: item 4	0.701^**^	0.441^*^	0.534^**^	0.555^**^		
NBS: item 5	0.795^**^	0.675^**^	0.701^**^	0.670^**^	0.436^*^	
PANSS: delusion item	0.672^**^	0.639^**^	0.594^**^	0.573^**^	0.472^*^	0.598^**^
PDI: number of “YES” items	0.511^**^	0.561^**^	0.397^*^	0.461^*^	0.306	0.482^**^
PDI: preoccupation	0.482^**^	0.521^**^	0.406^*^	0.409^*^	0.333	0.388^*^
PDI: conviction	0.545^**^	0.576^**^	0.457^*^	0.492^**^	0.349	0.459^*^
PDI: distress	0.486^**^	0.562^**^	0.424^*^	0.438*	0.230	0.425^*^
BCIS: total score	0.128	0.190	0.128	0.213	−0.053	0.059
BCIS: self-reflectiveness	−0.102	0.022	−0.030	−0.021	−0.178	−0.289
BCIS: self-certainty	0.289	0.237	0.209	0.316	0.123	0.400^*^
PHQ-9	0.197	0.371	0.253	0.126	−0.175	0.279
GAD-7	0.359	0.492^**^	0.401^*^	0.252	0.000	0.401^*^

^**^p < 0.01, ^*^p < 0.05.

**Figure 2:**
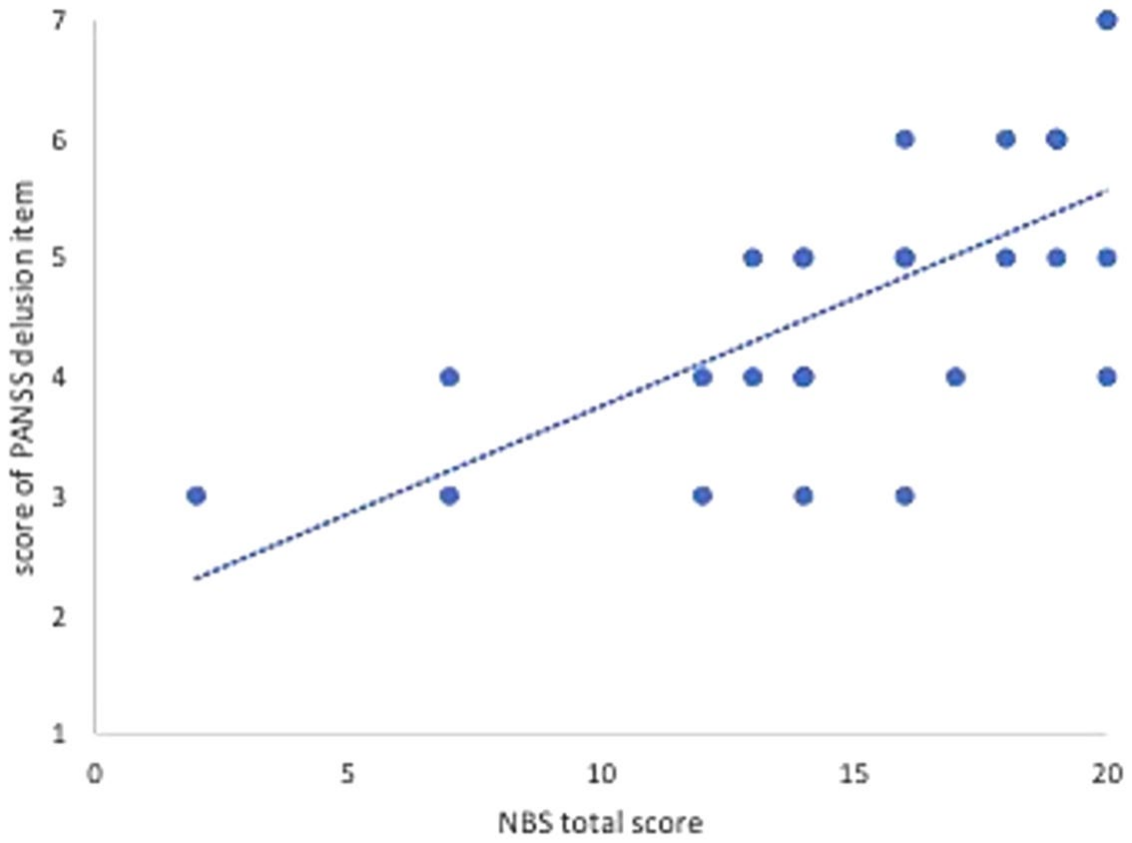
Correlations between NBS total score and score of PANSS delusion item.

The correlation between the total score of the NBS and the score of the PANSS delusion item as other objective rating scales is shown in Figure 2, because there is no Japanese version of the BABS.

Following the findings of Barton et al. (4), the NBS total score was classified into three levels: 0–10: “good/fair insight,” 11–15: “overvalued idea,” and 16–20: “delusional,” with four respondents having “good/fair insight,” nine having “overvalued idea,” and 15 having “delusional.”

The alpha coefficient of the NBS total score from the first evaluation by Rater 1 was 0.902. The mean of total NBS score from the second evaluation by Rater 1 was 15.29 (SD = 4.45). The intraclass correlation coefficient ICC (1.2) for the retest was 0.903 for total score, 0.863 for “conviction,” 0.776 for “fixity,” 0.878 for “fluctuation,” 0.749 for “resistance,” and 0.794 for “reasonableness of belief “(all *p* < 0.001).

The mean total NBS score as rated by Rater 2 was 14.21 (SD = 4.22). The ICC (2.2) between raters was 0.853 (*p* < 0.001) for total score, 0.754 (*p* < 0.001) for “conviction,” 0.724 (*p* < 0.001) for “fixity,” 0.836 (*p* < 0.001) for “fluctuation,” 0.664 (*p* = 0.003) for “resistance,” and 0.702 for “reasonableness of beliefs” (*p* = 0.001).

A rating for ease of response did not correlate significantly with other measures, including the NBS. The “resistance” of NBS and “self-certainty” of BCIS had negative correlations with this rating, but they were not significantly.

Table 2 shows the correlations between the NBS scores and each scale. There were significant correlations with the NBS total score and the PANSS delusion items, the number of “yes” items on the PDI-21, and the level of preoccupation, conviction, and distress, but not with the BCIS total score and subscales, PHQ-9, and GAD-7. The correlation coefficients between BCIS total score, self-reflectiveness, and self-confidence were 0.656 (*p* < 0.001) for total/self-reflectiveness, 0.716 (p < 0.001) for total/self-confidence, and − 0.057 for self-reflectiveness/self-confidence. The correlation between total BCIS and PDI-21 total scores was 0.471 (*p* = 0.011).

## 4 Discussion

NBS has been used to measure pathological beliefs in obsessive-compulsive disorder, acutely hospitalized psychosis, and anorexia nervosa patients, and has been found to be reliable and valid. This study was designed to confirm the feasibility of using NBS for assessing pathological beliefs in patients with chronic-phase schizophrenia during outpatient visits.

All the participants were diagnosed with SCID-5-RV schizophrenia. The mean duration of illness was more than 25 years, and the condition was considered chronic; the mean PANSS delusion item score suggested that participants had mild to moderate symptoms; the mean number of items answered “yes” on the PDI-21 was 8; the mean score of psychosis on the PDI-21 developed by Peters et al. (8) who developed the PDI-21, was 11.9, but all participants were hospitalized. Based on the results of the PANSS, PDI-21, and NBS, participants were those who were receiving ongoing treatment but had thoughts and beliefs that could be considered overvalued ideas or delusions.

The mean NBS total score in acute hospitalized psychosis in Rajendran et al. (5) was 15.99, and in anorexia nervosa in Barton et al. (4) was 14.30; therefore, the present mean of 14.93 is reasonable compared to these previous studies. In Barton et al. (4), six participants had “good/fair insight,” 10 participants had “overvalued idea,” and five participants were “delusional,” whereas in the present study, in order, four, nine, and 15, and the proportion of “delusional” among the number of participants was high. This also seems to be a reasonable result given the differences in disorders. These results suggest the validity of NBS for chronic-phase schizophrenia from a clinical perspective.

Owing to its high alpha coefficient, the NBS is considered a coherent and reliable instrument. In terms of the number of intraclass correlations, the retest reliability was higher than that reported by (5), while the inter-rater reliability was slightly lower. The reason for the higher retest reliability in the present study may be that the participants in the earlier study were inpatients in acute wards, their treatment was successful, and their overall condition had improved by the time of the second survey. Conversely, the delusional beliefs of patients with chronic schizophrenia persisted relatively stable.

The reasons why inter-rater reliability was somewhat lower in this study than in previous studies can be explained as follows. The two hospitals where the study was conducted were geographically distant (approximately 1,040 km). Although the research meetings were conducted online, the staff of the two hospitals had no experience in collaborating in clinical or research studies. Therefore, a practice video was created to align the raters’ proficiency levels, and they were trained prior to the survey. This was effective; and the results were statistically sound; however, the evaluations may not have been as consistent as those between members who worked together on many projects.

The NBS correlated well with the PANSS delusion item, an objective assessment of delusion, and PDI-21, a self-administered scale. However, correlations with the PHQ-9 and GAD-7, which measure depression and anxiety respectively, were not significant. These results suggest that the construct validity of the NBS was adequate; and that the NBS has high potential for use in measuring beliefs in chronic schizophrenia, as in the present study.

Cognitive impairment may be more severe in chronic schizophrenia than in other psychiatric disorders (14). Therefore, a scale that allows for a reliable survey in a short time and with a small number of items is clinically useful because it is less invasive for the interviewee and facilitates symptom assessment. In addition, although delusions often persist in chronic schizophrenia, the condition and its severity may shift over time, not as much as in acute cases (15), and a scale measuring only severe delusions may be inappropriate. As previous studies have shown, NBS is considered appropriate for examining various beliefs because it can examine a wide range of beliefs on the same scale, from obsessions and overvalued ideas to delusions in the acute phase.

Additionally, these results indicate that the targets for comparing pathological beliefs using NBS can be extended to chronic schizophrenia. We have been practicing the Metacognitive Training in Japan (16), and a meta-analysis demonstrated that this intervention improved delusions in schizophrenia (17, 18). However, the process of change of delusions is not yet known in detail. NBS may be useful for evaluating the effectiveness of such psychosocial interventions and for elucidating treatment mechanisms, and a project will be initiated to evaluate delusions in chronic-phase schizophrenia by NBS before and after the Metacognitive Training (JSPS Grant-in-Aid for Scientific Research, PI: Takuma Ishigaki).

It should be noted, however, that the mean rating score for ease of response was 1.21. Although the NBS is an easy-to-instruct and easy-to-respond scale, this score should have been closer to 2 if no additional explanation was needed for the participants. The present results suggest that to obtain reliable responses to the questions from at least middle-aged Japanese people with chronic schizophrenia, it is necessary to explain the meaning of the words used in the questions and provide some examples. Conversely, if such explanations are included, reliable responses can be obtained, as in the present study.

No association was found with insights into the disease as measured by the BCIS. The Japanese version of the BCIS showed sufficient internal consistency and reasonable correlation with the PDI-21, suggesting that the scale itself was not problematic. Further studies are needed to investigate the relationship between the NBS and the insight of diseases.

### 4.1 Limitations of the present study

In the near future, our research group plans to compare delusions in chronic schizophrenia with overvalued ideas or delusional beliefs in other mental disorders using NBS. We expect to eventually obtain data on approximately 50 subjects in each group; however, the number of subjects in this study was small. This is a preliminary study. However, as this study suggests the high validity and reliability of the NBS, we plan to proceed toward the final objective.

The Brown Assessment of Beliefs Scale and Overvalued Ideas Scale, which have been used in previous studies to confirm the validity of the NBS, do not have Japanese versions. Therefore, in this study, the delusion item of the PANSS, an objective scale, and the PDI-21, a self-administered scale, were used to confirm construct validity. However, criterion-related validity could not be verified.

The current study did not measure participants’ cognitive abilities. It is assumed that there is variability in such abilities in chronic schizophrenia (14). Difficulty in understanding or answering questions may be related to these and should be examined closely in the future.

## 5 Conclusion

This study showed that NBS is both reliable and valid when used in patients with chronic schizophrenia, provided that the assessor explains the questions carefully. Based on the NBS results, patients with chronic schizophrenia may have delusions and beliefs that belong to overvalued ideas. It is suggested that in the future, NBS may be useful not only for psychopathological comparisons of delusions and overvalued ideas, but also for long-term prognostic studies with therapeutic interventions such as cognitive behavioral therapy.

## Data availability statement

The raw data supporting the conclusions of this article will be made available by the authors, without undue reservation.

## Ethics statement

The studies involving humans were approved by The Committee on Ethics of Experimental Research on Human Subjects Graduate School of Arts and Sciences/College of Arts and Sciences, The University of Tokyo. The studies were conducted in accordance with the local legislation and institutional requirements. The participants provided their written informed consent to participate in this study.

## Author contributions

TI: Conceptualization, Data curation, Formal analysis, Funding acquisition, Methodology, Project administration, Resources, Software, Validation, Visualization, Writing – original draft, Writing – review & editing. TS: Data curation, Investigation, Supervision, Writing – review & editing. HT: Data curation, Investigation, Supervision, Writing – review & editing. NY: Data curation, Investigation, Supervision, Writing – review & editing. YN: Formal analysis, Methodology, Supervision, Writing – review & editing. RI: Formal analysis, Methodology, Supervision, Writing – review & editing. MH: Investigation, Supervision, Writing – review & editing.

## References

[1] Brakoulias V Starcevic V Milicevic D Hannan A Viswasam K Brown C. The Nepean belief scale: preliminary reliability and validity in obsessive–compulsive disorder. Int J Psychiatry Clin Pract. (2018) 22:84–8. doi: 10.1080/13651501.2017.1374413 28885070

[2] Eisen JL Phillips KA Baer L Beer DA Atala KD Rasmussen SA. The Brown assessment of beliefs scale: reliability and validity. Am J Psychiatry. (1998) 155:102–8. doi: 10.1176/ajp.155.1.102 9433346

[3] Neziroglu F McKay D Yaryura-Tobias JA Stevens KP Todaro J. The overvalued ideas scale: development, reliability and validity in obsessive-compulsive disorder. Behav Res Ther. (1999) 37:881–902. doi: 10.1016/S0005-7967(98)00191-0 10458051

[4] Barton R Aouad P Hay P Buckett G Russell J Sheridan M. Distinguishing delusional beliefs from overvalued ideas in anorexia nervosa: an exploratory pilot study. J Eat Disord. (2022) 10:85. doi: 10.1186/s40337-022-00600-2, PMID: 35739570 PMC9229879

[5] Rajendran P Winssen CV Viswasam K Tariq N Espinoza D Starcevic V. The psychometric properties of the Nepean belief scale as a tool for assessing delusions in schizophrenia and related psychotic disorders. Compr Psychiatry. (2022) 117:152337. doi: 10.1016/j.comppsych.2022.152337 35863256

[6] First MB Williams JBW Karg RS Spitzer RL. Structured clinical interview for DSM-5 disorders SCID-5-RV. Washington D.C: American Psychiatry Association (2015) (translated by Takahashi, S. and Kitamura, T. (2020). Tokyo: Igaku-shoin).

[7] Kay S.R. Opler L.A. Fiszbein A. (1991). Positive and negative syndrome scale (PANSS) rating manual. Multi-health Inc. (translated by Yamada, H., Masui, K., Kikumoto, K. (1991) Tokyo: Seiwa-shoten). 10.1093/schbul/13.2.261 3616518

[8] Peters E Joseph S Day S Garety P Garety P. Measuring delusional ideation: the 21-item Peters et al. delusions inventory (PDI). Schizophr Bull. (2004) 30:1005–22. doi: 10.1093/oxfordjournals.schbul.a007116 15954204

[9] Yamasaki S Arakawa H Tanno Y. Delusion-like ideation and coping: avoidant vs. planning problem-solving coping strategy. Jpn. J. Per. (2006) 14:254–65. doi: 10.2132/personality.14.254

[10] Beck AT Baruch E Balter JM Steer RA Warmanet DM. A new instrument for measuring insight: the Beck cognitive insight scale. Schizophr Res. (2004) 68:319–29. doi: 10.1016/S0920-9964(03)00189-0, PMID: 15099613

[11] Uchida T Matsumoto K Kikuchi A Miyakoshi T Ito F Ueno T. Psychometric properties of the Japanese version of the Beck cognitive insight scale: relation of cognitive insight to clinical insight. Psychiatry Clin Neurosci. (2009) 63:291–7. doi: 10.1111/j.1440-1819.2009.01946.x, PMID: 19566759

[12] Spitzer RL Kroenke K Williams JB. Validation and utility of a self-report version of PRIME-MD: the PHQ primary care study. Primary care evaluation of mental disorders. Patient health questionnaire. JAMA. (1999) 282:1737–44. doi: 10.1001/jama.282.18.1737, PMID: 10568646

[13] Spitzer RL Kroenke K Williams JB Loewe B. A brief measure for assessing generalized anxiety disorder: the GAD-7. Arch Intern Med. (2006) 166:1092–7. doi: 10.1001/archinte.166.10.1092 16717171

[14] Sheffield JM Karcher NR Barch DM. Cognitive deficits in psychotic disorders: a lifespan perspective. Neuropsychol Rev. (2018) 28:509–33. doi: 10.1007/s11065-018-9388-2, PMID: 30343458 PMC6475621

[15] Harow M Jobe TH. How frequent is chronic multilayer delusional activity and recovery in schizophrenia: a 20-year multi-follow-up. Schizophr Bull. (2010) 36:192–204. doi: 10.1093/schbul/snb074, PMID: 18617485 PMC2800138

[16] Ishikawa R Ishigaki T Shimada T Tanoue H Yoshinaga N Oribe N. The efficacy of extended metacognitive training for psychosis: a randomized controlled trial. Schizophr Res. (2020) 215:399–407. doi: 10.1016/j.schres.2019.08.006, PMID: 31471248

[17] Moritz S Veckenstedt R Bohn F Hottenrott B Scheub F Randjbar S. Complementary group metacognitive training (MCT) reduces delusional ideation in schizophrenia. Schizophr Res. (2013) 151:61–9. doi: 10.1016/j.schres.2013.10.007, PMID: 24183707

[18] Sauvé G Lavignea KM Pochiet G Brodeur MB Lepage M. Efficacy of psychological interventions targeting cognitive biases in schizophrenia: a systematic review and meta-analysis. Clin Psychol Rev. (2020) 78:101854. doi: 10.1016/j.cpr.2020.101854, PMID: 32361339

